# Cleansing the Oral Cavity

**Published:** 1903-12

**Authors:** S. Blair Luckie

**Affiliations:** Chester, PA.


### CLEANSING THE ORAL CAVITY.

BY S. BLAIR LUCKIE, D.D.S., CHESTER, PA.

To secure and maintain a healthy condition of the human mouth often requires strenuous efforts on the part of both the patient and the dentist.

A condition of health, or one approximating it, is obtained by frequent visits for the removing of collections from those places inaccessible to the brush, or difficult of removal by other means usually employed by the patient; it is, however, accompanied by inconvenience and expense, and places the responsibility largely upon the dentist.

It is well, at least in those cases requiring absolute cleanliness, to place the responsibility of maintaining the condition antagonistic to disease as much as possible upon the patient. Especially in pyorrhœa alveolaris and gingivitis should the patient be impressed with the fact that cure and its maintenance must largely depend upon his vigilance.

For some time I have been recommending to those of my patients who are affected with pyorrhœa alveolaris and gingivitis a douching of the mouth by means of a rubber hose attached to the faucet of a bath-tub. The modern tub being supplied with a double faucet makes it convenient to obtain a force of water at the proper temperature. The force and temperature is under the control of the patient. By the pressure of the hose between thumb and index-finger the force can be increased to a degree sufficient to expel from the interstitial spaces all deposits that are not attached to the teeth.

All the surfaces of the oral cavity can be subjected to the action of the stream,—the gums, buccal surfaces, under the tongue, the teeth, and, to those persons not easily gagged, the fauces.

During the operation the mouth is kept open, allowing the water to flow into the tub.

The results obtained from this treatment make it worthy to be presented to the profession. Not only is it an effectual way of cleansing the oral cavity, but the force of the stream seems to have an effect upon the gums similar to massage, causing the product of disease to be removed.

